# Immune-related Adverse Events after Immune Checkpoint Blockade–based Therapy Are Associated with Improved Survival in Advanced Sarcomas

**DOI:** 10.1158/2767-9764.CRC-22-0140

**Published:** 2023-10-18

**Authors:** Evan Rosenbaum, Kenneth Seier, Martina Bradic, Ciara Kelly, Sujana Movva, Benjamin A. Nacev, Mrinal M. Gounder, Mary L. Keohan, Viswatej Avutu, Ping Chi, Katherine A. Thornton, Jason E. Chan, Mark A. Dickson, Mark T.A. Donoghue, William D. Tap, Li-Xuan Qin, Sandra P. D'Angelo

**Affiliations:** 1 Department of Medicine, Memorial Sloan Kettering Cancer Center, New York, New York.; 2 Department of Medicine, Weill Cornell Medical College, New York, New York.; 3 Department of Epidemiology and Biostatistics, Memorial Sloan Kettering Cancer Center, New York, New York.; 4 Marie-Josée and Henry R. Kravis Center for Molecular Oncology, Memorial Sloan Kettering Cancer Center, New York, New York.; 5 Human Oncology and Pathogenesis Program, Memorial Sloan Kettering Cancer Center, New York, New York.

## Abstract

The association between immune-related AEs (irAE) and outcome in patients with sarcoma is not known. We retrospectively reviewed a cohort of patients with advanced sarcoma treated with immune checkpoint blockade (ICB)-based therapy. Association of irAEs with survival was assessed using a Cox regression model that incorporated irAE occurrence as a time-dependent covariate. Tumor samples with available RNA sequencing data were stratified by presence of an irAE to identify patterns of differential gene expression. A total of 131 patients were included. Forty-two (32%) had at least one irAE of any grade and 16 (12%) had at least one grade ≥ 3 irAE. The most common irAEs were hypothyroidism (8.3%), arthralgias (5.3%), pneumonitis (4.6%), allergic reaction (3.8%), and elevated transaminases (3.8%). Median progression-free survival (PFS) and overall survival (OS) from the time of study entry were 11.4 [95% confidence interval (CI), 10.7–15.0) and 74.6 weeks (CI, 44.9–89.7), respectively. On Cox analysis adjusting for clinical covariates that were significant in the univariate setting, the HR for an irAE (HR, 0.662; CI, 0.421–1.041) approached, but did not reach statistical significance for PFS (*P* = 0.074). Patients had a significantly lower HR for OS (HR, 0.443; CI, 0.246–0.798; *P* = 0.007) compared with those without or before an irAE. Gene expression profiling on baseline tumor samples found that patients who had an irAE had higher numbers of tumor-infiltrating dendritic cells, CD8^+^ T cells, and regulatory T cells as well as upregulation of immune and inflammatory pathways.

**Significance:**

irAE after ICB therapy was associated with an improved OS; it also approached statistical significance for improved PFS. Patients who had an irAE were more likely to have an inflamed tumor microenvironment at baseline.

## Introduction

Immune checkpoint blockade (ICB) with anti-programmed cell death protein 1 (PD-1) antibody therapy is associated with a unique set of toxicities thought to arise from activation of CD8^+^ T cells that are reactive to self-antigens (1). The most frequent of these immune-related adverse events (irAE) are pruritis, rash, diarrhea, colitis, hypothyroidism, hyperthyroidism, and pneumonitis (2). Approximately 10% of irAEs secondary to ICB therapy are grade ≥ 3, while the incidence is higher (36%) with ICB combinations (3, 4).

Early studies of ICB in melanoma identified an association between immune-related toxicity and study endpoints indicative of clinical benefit, such as objective response and overall survival (OS; refs. 5, 6). Subsequent investigations have confirmed this association in larger cohorts of carcinomas, such as urothelial carcinoma and non–small cell lung cancer (7–9). To our knowledge, this association has not been previously demonstrated in mesenchymal neoplasms. To date, there are no clinically validated biomarkers to predict the incidence of irAEs, although preliminary studies have identified the gut microbiome (10, 11) and a change in circulating immune cell subsets (12–14) as factors associated with the likelihood of developing an irAE.

To determine whether the development of an irAE is associated with clinical benefit in sarcomas, we reviewed the incidence of irAEs across sarcoma-specific ICB-based clinical trials at our institution and correlated their occurrence with clinical outcome. Gene expression profiles of baseline tumor biopsies were examined for differences between patients with or without an irAE to identify potential patterns that may be predictive of toxicity to ICB-based therapies in sarcoma.

## Materials and Methods

### Patient Selection

Patients with unresectable or metastatic sarcoma treated at Memorial Sloan Kettering Cancer Center on one of three phase II clinical trials of ICB-based therapy were eligible for inclusion in this retrospective analysis. The trials included: nivolumab plus bempegaldesleukin (IL2 agonist), pembrolizumab plus epacadostat (indoleamine 2,3-dioxygenase 1 inhibitor), and pembrolizumab plus talimogene laherparepvec (modified oncolytic herpes simplex virus-1; NCT03282344, NCT03414229, and NCT03069378, respectively). The objective of this study was to pool the safety data from each trial, identify irAEs, and correlate them with best overall response by RECIST 1.1, progression-free survival (PFS) and OS. Adverse events (AE) were retrospectively designated as immune-related if the AE met previously published criteria (7) or if the AE was grade ≥ 2 with a likely immune basis resulting in dose modification, hospitalization, or corticosteroid use. Grade ≥ 2 hypothyroidism was considered an irAE.

### Statistical Analyses of Patient Outcomes

Summary statistics, median and range, were used to describe continuous variables and count and percent for categorical variables. OS was defined from time of trial entry until death (due to any cause) or last follow-up. PFS was defined from trial entry until progression, death or last follow-up. Univariate and multivariate Cox proportional hazards models were used to analyze OS and PFS outcomes. At the time of trial entry, AEs were not known to estimate the effect and measure the association between AEs and survival outcomes; therefore, they are included in the models as a time-dependent covariate (15). Three patients were identified who were treated on two of the included clinical trials. For these patients, their first entry date was used as the start of OS and PFS and they were censored at the time they entered the second trial. SAS version 9.4 SAS institute Inc. was used for all analyses. All tests were two sided and *P* < 0.05 was considered significant.

### Gene Expression Analyses

All patients who successfully underwent tumor biopsy per study protocol at the prespecified baseline timepoint (prior to initiation of study therapy) were screened for inclusion in this analysis. In total, tumor samples from 71 unique patients successfully underwent whole-transcriptome sequencing. After RiboGreen quantification and quality control by Agilent BioAnalyzer, 469–500 ng of total RNA with RNA integrity values of 6.8–10 underwent polyA selection and TruSeq library preparation according to instructions provided by Illumina (TruSeq Stranded mRNA LT Kit, catalog no. RS-122-2102), with 8 cycles of PCR. Samples were barcoded and run on a HiSeq 4000 in a PE100 run, using the HiSeq 3000/4000 SBS Kit (Illumina). An average of 41 million paired reads was generated per sample. Ribosomal reads represented 0.9%–5.9% of the total reads generated and the percent of mRNA bases averaged 64%. Fastq files were processed using an in-house RNA sequencing pipeline. Briefly, reads were aligned to GRCh37.75 human reference using STAR v2.7.0f (16) and Ensembl v75. (17, 18). Picard v. 2.22.0 (19) was used to perform quality control of the bam files resulting from alignment. Kallisto v0.46.2 (20) was used for expression quantification and gene level expression was summarized based on Enembl v75. Normalized TPM were computed using Sleuth v0.30 (sleuth_to_martix).

To determine significantly expressed genes, we used the Wald test. *P* values were corrected using the Benjamini and Hochberg (BH) method (21). A gene was considered differentially expressed if *q* < 0.05. Pathway enrichment analysis was performed on 50 Hallmark pathways downloaded from MSigDB (https://www.gsea-msigdb.org/gsea/msigdb/). R package clusterProfiler and the GSEA function in R were used in gene set enrichment analysis (GSEA; refs. 22, 23). Genes were ranked by the effect size, which was calculated by multiplying *P* value (*P* value based on comparison between two conditions: presence and absence of irAE) with the sign of log-fold change. These values where then used for the ranking of the genes. Weighted enrichment statistics and 100,000 gene set permutations were subsequently calculated and used for detection of significantly enriched pathways. We quantified immune cell populations using the immunedeconv R package and quanTIseq function 3.6.3 (24, 25). Difference in cell proportion between irAE and non-irAE were calculated using a linear model, where gene expression represented the dependent variable, and irAE presence represented the independent variable. Sarcoma histology and technical batches related to sequencing were included as covariates in the model (model: gene expression ∼ irAE + histology + batch). *P* values were corrected using the BH method. Hierarchical clustering of samples (i.e., maximum clustering distance) was then conducted on the basis of these Z-scores of immune cell proportions. Z-scores for all samples (z = (*x* − µ)/σ), where *x* is the raw cell fraction, µ is all samples mean, and σ is the SD, were values used for the hierarchical clustering.

### Data Availability

All sequencing data, where informed consent has been obtained from the patient, is publicly available at The database of Genotypes and Phenotypes (dbGaP: phs003284), accession number PRJNA962616 and ID number 962616. Seven samples are not publicly available due to lack of consent for their release.

## Results

A total of 131 patients across three trials were included (Table 1; Supplementary Table S1). Forty-two patients (32%) had at least 1 irAE of any grade and 16 (12%) had at least one grade ≥ 3 irAE. The irAEs experienced across studies are listed in Table 2. The most common were hypothyroidism (8.4%), arthralgias (5.3%), pneumonitis (4.6%), and allergic reaction (3.8%). The most frequent grade ≥ 3 irAEs were pneumonitis (2.3%), pancreatitis, elevated transaminases, elevated amylase, and arthritis (1.5% each).

**TABLE 1:** Patient characteristics

	Total (*n* = 131)
Age	
Median (IQR)	56.0 (43.0–66.0)
Gender	
F	63 (48.1%)
M	68 (51.9%)
Histologic subtype	
Leiomyosarcoma	19 (14.5%)
Angiosarcoma	14 (10.7%)
Dedifferentiated liposarcoma	12 (9.2%)
Osteosarcoma	11 (8.4%)
Other^a^	60 (45.8%)
Clinical trial	
Nivolumab plus bempegaldesleukin	83 (63.4%)
Pembrolizumab plus epacadostat	28 (21.4%)
Pembrolizumab plus TVEC	20 (15.3%)
Patients with ≥ 1 irAE	
Grade < 3	26 (31.6%)
Grade ≥ 3	16 (12.0%)
Baseline ECOG performance status	
0	91 (69.5%)
1	39 (29.8%)
2	1 (00.8%)
Best overall response by RECIST 1.1	
NE	4 (3.1%)
PD	69 (52.3%)
PR	16 (12.2%)
SD	42 (32.1%)
Prior lines of systemic therapy	
Median (IQR)	2.0 (1.0–4.0)
<4	94 (71.8%)
≥4	37 (28.2%)

Abbreviations: ECOG, Eastern Cooperative Group; IQR, interquartile range; irAE, immune-related adverse event; NE, not evaluable; PD, progressive disease; PR, partial response; SD, stable disease.

^a^A complete list of histologic subtypes is presented in Supplementary Table S1.

**TABLE 2:** irAEs seen across trials

	Grade 1–2		Grade ≥3		Any grade	
irAE	*n*	%	*n*	%	*n*	%
Hypothyroid	11	8.40	0	0.00	11	8.40
Arthralgia	6	4.58	1	0.76	7	5.34
Pneumonitis	3	2.29	3	2.29	6	4.58
Allergic reaction	5	3.82	0	0.00	5	3.82
AST/ALT Increased	3	2.29	2	1.53	5	3.82
Rash	3	2.29	1	0.76	4	3.05
Infusion reaction	4	3.05	0	0.00	4	3.05
Arthritis	1	0.76	2	1.53	3	2.29
Pancreatitis	0	0.00	2	1.53	2	1.53
Amylase increased	0	0.00	2	1.53	2	1.53
AKI	0	0.00	1	0.76	1	0.76
Colitis	0	0.00	1	0.76	1	0.76
Diarrhea	0	0.00	1	0.76	1	0.76
Lipase increased	0	0.00	1	0.76	1	0.76
Parotitis	0	0.00	1	0.76	1	0.76
Bilirubin Increased	1	0.76	0	0.00	1	0.76
Peripheral motor neuropathy	1	0.76	0	0.00	1	0.76
Peripheral sensory neuropathy	1	0.76	0	0.00	1	0.76
Thrombocytopenia	1	0.76	0	0.00	1	0.76
Uveitis	1	0.76	0	0.00	1	0.76
Ptosis	1	0.76	0	0.00	1	0.76
Myalgia	1	0.76	0	0.00	1	0.76

Abbreviations: AST, aspartate aminotransferase; ALT, alanine aminotransferase; AKI, acute kidney injury.

NOTE: All *n* values represent number of patients.

**FIGURE 1:**
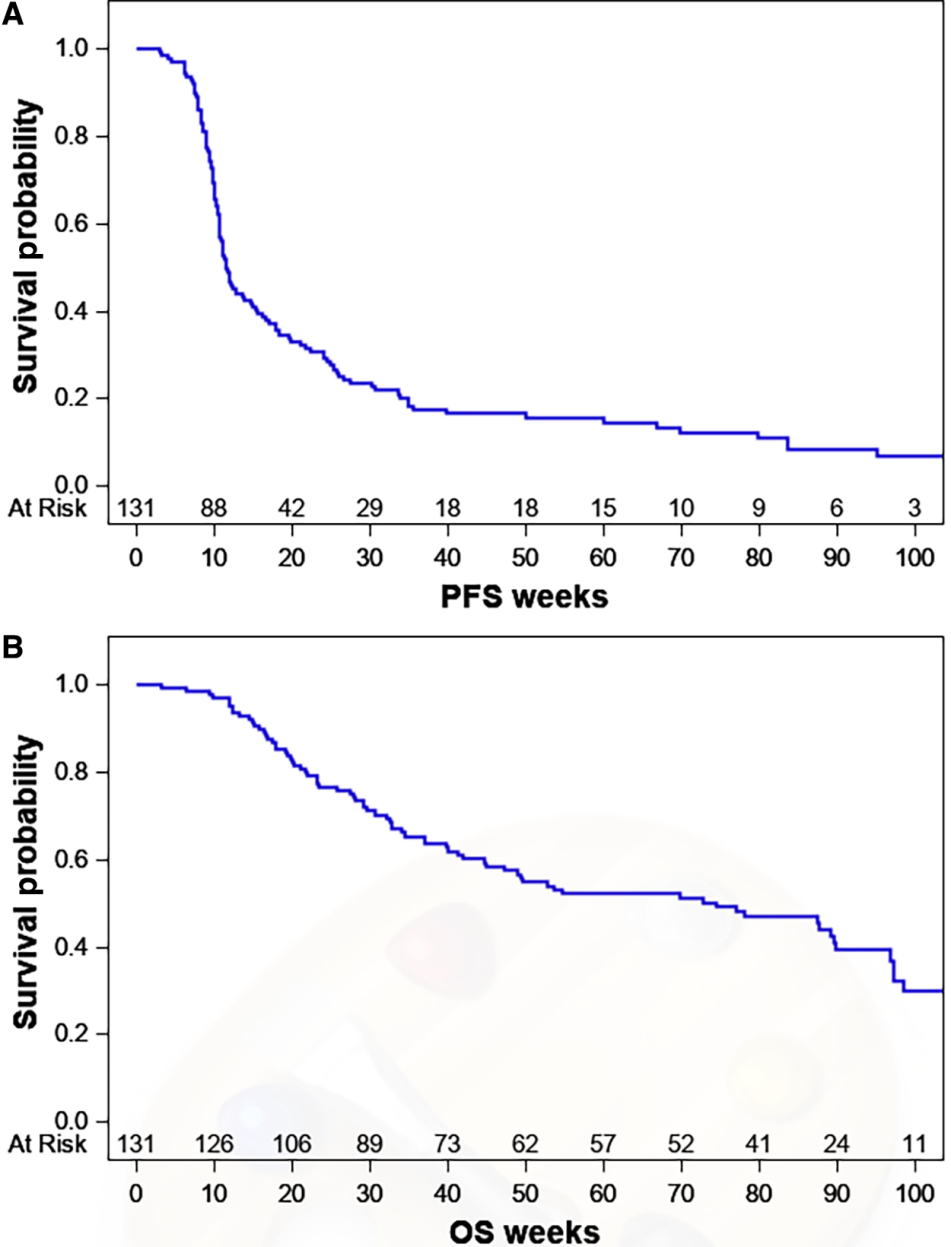
Kaplan–Meier plots of PFS (**A**) and OS (**B**) in 131 patients treated on ICB-based trials.

An objective response was seen in 16 patients: 7 patients with undifferentiated spindle and/or pleomorphic sarcoma, 4 with angiosarcoma, 2 with leiomyosarcoma, and 1 each with alveolar soft part sarcoma, dedifferentiated chondrosarcoma, and epithelioid sarcoma. Among responders, 11 (68.8%) had at least one irAE of any grade and 4 (25%) had a grade ≥ 3 irAE. The median PFS from the time of study entry across all patients was 11.4 weeks [95% confidence interval (CI) 10.7–15.0; Fig. 1A]. On univariate analysis, younger age and higher baseline Eastern Cooperative Group performance status were significantly associated with a shorter PFS (Table 3). On univariate PFS analysis, the HR for an irAE (HR, 0.678; 95% CI, 0.432–1.065; *P* = 0.091) did not reach the statistical significance threshold. On multivariate analysis, performance status (HR, 1.749; 95% CI, 1.172–2.611; *P* = 0.006) remained statistically significant, while age (HR, 0.989; 95% CI, 0.979–1.00; *P* = 0.056) and development of irAE (HR, 0.662; 95% CI, 0.421–1.041; *P* = 0.074) approached statistical significance indicating a possible association between these variables and longer PFS.

**TABLE 3:** PFS and OS analyses across three trials utilizing immune checkpoint inhibition in sarcoma

			Univariate analysis				Multivariate analysis		
	Variable	Reference value	HR	95% CI	*P*	Reference value	HR	95% CI	*P*
PFS	Age^a^		0.988	0.977–0.999	0.038^b^		0.989	0.979–1.000	0.056
	Baseline ECOG	0	1.705	1.147–2.535	0.008^b^	0	1.749	1.172–2.611	0.006^b^
	Gender	M	0.934	0.645–1.354	0.719				
	Prior systemic therapy	≤3	1.337	0.889–2.011	0.162				
	irAE^c^		0.678	0.432–1.065	0.091		0.662	0.421–1.041	0.074
OS	Age^a^		0.993	0.980–1.007	0.341				
	Baseline ECOG	0	1.610	1.996–2.603	0.052	0	1.418	0.853–2.359	0.178
	Gender	M	0.882	0.557–1.396	0.591				
	Prior systemic therapy	≤3	1.522	0.938–2.469	0.089	≤3	1.334	0.800–2.226	0.269
	PD^c^		3.069	1.891–4.979	<0.001^b^				
	PR^c^		0.307	0.111–0.850	0.023^b^				
	SD^c^		0.474	0.274–0.819	0.007^b^				
	irAE^c^		0.434	0.241–0.782	0.005^b^		0.443	0.246–0.798	0.007^c^

Abbreviations: CI, confidence interval; ECOG, Eastern Cooperative Group; HR, hazard ratio; irAE, immune-related adverse event; PD, progressive disease; PR, partial response; SD, stable disease.

^a^Continuous variable.

^b^Signifies *P* < 0.05.

^c^Time-dependent variable.

Median OS across the studies was 74.6 weeks (95% CI, 44.9–89.7; Fig. 1B). On univariate analysis, development of an irAE was associated with better OS (HR, 0.434; 95% CI, 0.241–0.782; *P* = 0.005; Table 3). Interestingly, patients who achieved an objective response or stable disease by RECIST 1.1 had a statistically significant decrease in the HR for death (HR, 0.307; 95% CI, 0.111–0.850; *P* = 0.023 and HR, 0.474; 95% CI, 0.274–0.819; *P* = 0.007, respectively). This contrasts with patients who had progressive disease, whose HR for death after RECIST-defined progression was significantly higher (HR, 3.069; 95% CI, 1.891–4.979; *P* ≤ 0.001). On multivariate analysis, while controlling for baseline performance status and number of prior lines of systemic therapy, patients had a significantly lower HR for OS after experiencing an irAE (HR, 0.443; 95% CI, 0.246–0.798; *P* = 0.007).

**FIGURE 2:**
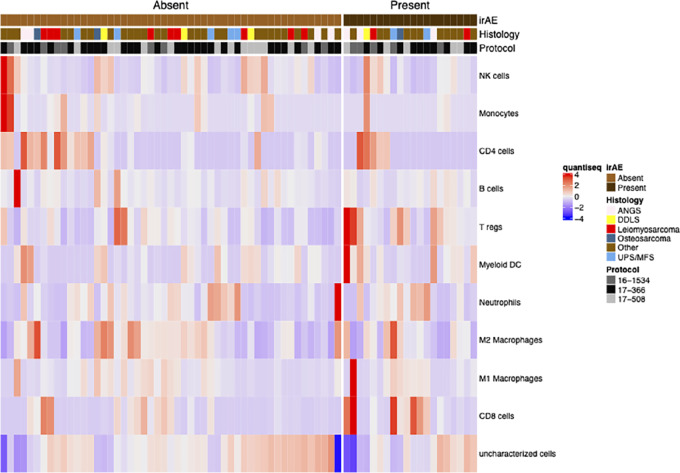
Tumor immune microenvironments in patients with and without irAE: Heat map of immune cell fractions estimated using quanTIseq for immune deconvoluation clustered by irAE status.

**FIGURE 3:**
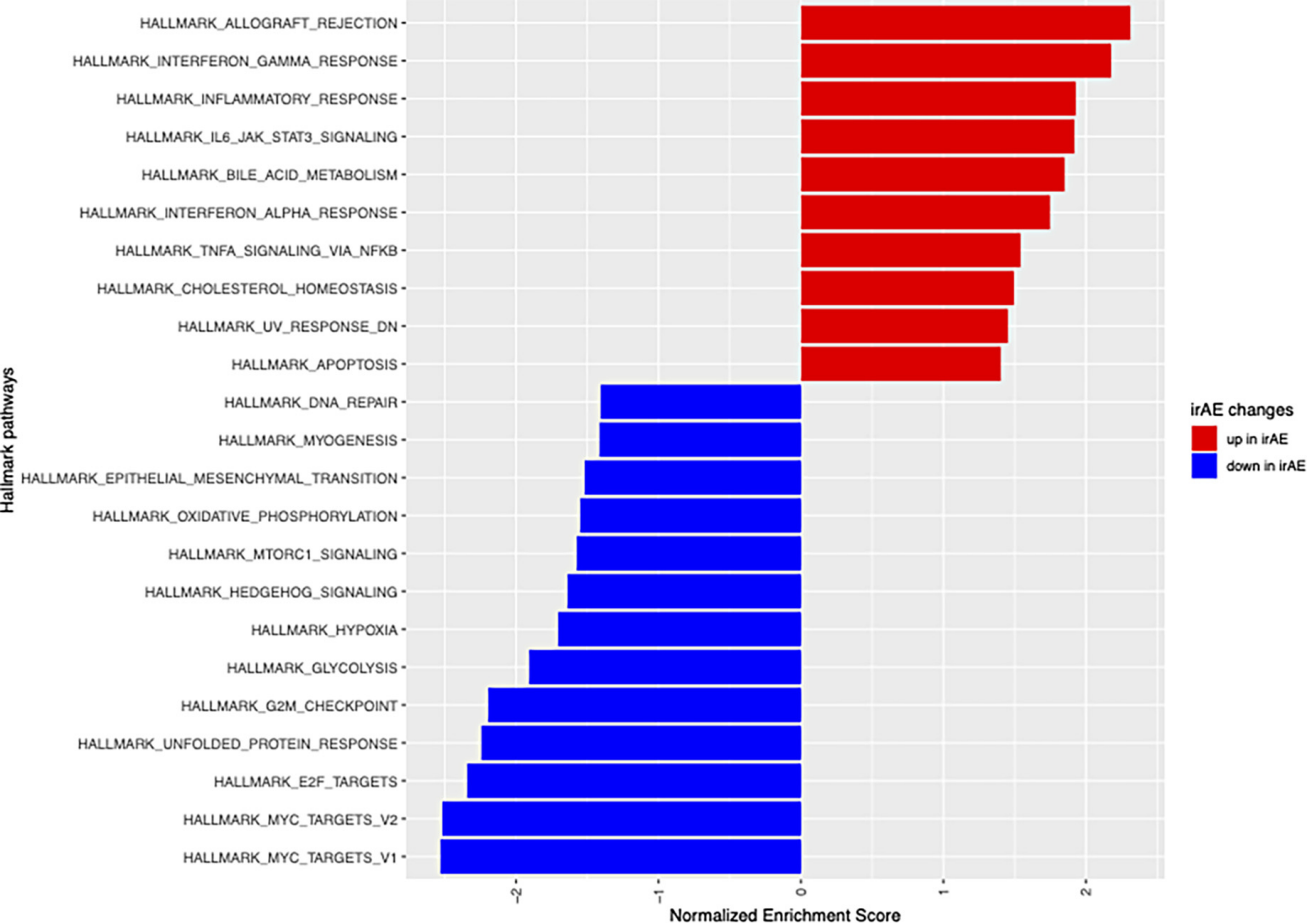
The results from GSEA with the gene set names from Hallmark pathways and the normalized enrichment scores shown. Positive enrichment score represents pathways associated with presence of irAE (red bars), while negative enrichment score (blue bars) represents those that were associated absence of irAE. All Hallmark pathways shown had an adjusted *P* value < 0.05.

A total of 71 pretreatment samples were transcriptionally profiled, of which 20 had an irAE (Supplementary Table S2). Tumor immune cell populations were deconvoluted using quanTIseq (25) and the difference in immune cell expression between patients who had at least one irAE and patients without were compared in a multivariate model utilizing tumor histology and technical sequencing batch as covariates (Fig. 2). CD8^+^, regulatory T cells, and myeloid dendritic cells were significantly enriched in patients who had an irAE (BH *P* value < 0.05; Table 4). GSEA of Hallmark pathways found the following immune-related pathways were upregulated in patients who had an irAE: allograft rejection, IFNγ response, inflammatory response, JAK-STAT signaling, IFNα response, and TNFα signaling via NFκb. In contrast, MYC targets, E2F targets, G_2_–M checkpoint, hedgehog, and epithelial–mesenchymal transition pathways were downregulated in patients with an irAE (adjusted *P* value < 0.05; Fig. 3; Supplementary Table S3).

**TABLE 4:** Association between immune cell proportions in patients with and without irAE

Immune cell subset	*P* value	Adjusted *P* value^a^
B cells	0.66	0.819
M1 Macrophages	0.17	0.378
M2 Macrophages	0.67	0.819
Monocytes	0.40	0.625
Myeloid DC	0.00	0.016^a^
Neutrophils	0.96	0.961
NK cells	0.37	0.625
CD4 cells	0.86	0.941
CD8 cells	0.00	0.016^a^
Tregs	0.00	0.015^a^
Uncharacterized cells	0.16	0.378

^a^Benjamini-Hochberg adjusted *P* value, *P* < 0.05.

## Discussion

Although ICB is not standard of care in most sarcoma subtypes, progress is being made to identify patients with the best chance of response to these agents. To date, undifferentiated pleomorphic sarcomas, cutaneous angiosarcomas, and alveolar soft part sarcomas, among a few others, have elicited promising responses to ICB treatment (26–30). Clinical trials in sarcomas are actively investigating combinatorial approaches of ICB plus alternative immune modulators, in a bid to overcome primary and secondary resistance to ICB monotherapy. Therefore, understanding the incidence of irAEs and understanding their clinical context in sarcoma remains relevant. To our knowledge, this is the first report in sarcoma to link the development of an irAE after ICB treatment with improved outcome. This finding has implications for clinicians who may want to rechallenge patients who experience irAEs with ICB or who wish to counsel patients about potential implications of an ICB-related AE.

Most sarcomas have a low tumor mutational burden and high burden of somatic copy-number changes, as outlined in The Cancer Genome Atlas (31) and other analyses (32). Yet, a proportion of tumors clearly have tumor-infiltrating immune cells, including lymphocytes, dendritic cells, and myeloid cells (31). Numerous immune checkpoints, including PD-1, are also expressed, although their expression varies across histologies (33). While PD-L1 does appear to be expressed among responders to ICB, it does not appear necessary for response (34). Most recently, an immune-hot microenvironment as determined by whole-transcriptome sequencing and the presence of intratumoral tertiary lymphoid structures has been correlated with response to ICB in sarcoma (35). Further work is ongoing to identify other predictive biomarkers of response to ICB in soft-tissue sarcoma.

Our exploratory analysis of RNA sequencing from baseline tumor samples revealed a significant association between immune hot tumors (those infiltrated with myeloid dendritic cells, CD8^+^ T cells, or regulatory CD4^+^ T cells) and development of an irAE, even after accounting for histologic subtype. Consistent with this finding, patients who developed an irAE tended to have upregulation of pathways association with inflammation and immune response in their tumors at baseline, suggesting that irAE development is intrinsically linked to a preexisting inflamed tumor microenvironment and a preexisting host response. As next-generation sequencing technologies become more adept at predicting response to ICB-based therapy in sarcoma, it is important to consider the implications of “immune-hot” tumors for the development of potential toxicities, especially as combination immunotherapies are more likely to cause irAEs than anti-PD-1 treatment alone.

This analysis also reports clinical outcome for a relatively large cohort of patients with sarcoma treated prospectively with ICB-based therapy. We report a median PFS of approximately 12 weeks among a population of pretreated patients with advanced disease. This indicates that ICB-based therapy provides meaningful clinical benefit in a portion of this patient population. Historical controls demonstrate that a progression-free rate of 40% at 12 weeks is considered clinically meaningful for second-line therapy or beyond in advanced soft-tissue sarcoma (36). In addition, our finding that patients who experienced a best overall response of SD by RECIST 1.1 had an OS advantage suggests that ICB may provide significant benefit despite failure to achieve radiographic tumor shrinkage. This is consistent with previously work demonstrating that sarcomas can demonstrate pathologic responses to therapy despite a lack of radiographic change by RECIST 1.1 (37).

Strengths of this study include its use of prospectively collected data and utilization of a time-dependent analysis to minimize guarantee-time bias (38). Time-dependent analyses may minimize bias that may have arisen in a time-to-event analysis (15), in which patients who respond to ICB have longer exposure to treatment and may be more likely to develop irAEs. The retrospective assignment of immune relatedness to AEs and the heterogenous nature of these three trials, which included other immune modulating drugs in addition to ICBs, are potential limitations. Because alternative immunomodulators have known immune-induced toxicities, extrapolation of these data to patients treated with ICI monotherapy must be done with caution. However, because of the rare nature of this heterogenous group of diseases, it would be challenging to build a large database of patients treated with ICB monotherapy on prospective trials.

## Supplementary Material

Supplementary Data Supplemental Table 1: Histologic subtype of sarcoma sorted by frequency; Supplemental Table 2: Histologic subtype, irAE, and response status of patients included in RNA seq analysis; Supplemental Table 3: GSEA of hallmark pathways by irAE status
